# Bilateral Discoid Medial Menisci: A Case Report and Review of the Literature

**DOI:** 10.5435/JAAOSGlobal-D-20-00069

**Published:** 2020-08-03

**Authors:** Kenneth Lukas, Holly Livock, Ken Kontio, Sasha Carsen

**Affiliations:** From the University of Dublin, Trinity College, College Green, Dublin, Ireland (Mr. Lukas), and the CHEO (Children's Hospital of Eastern Ontario) Research Institute, Ottawa, ON, Canada (Ms. Livock, Dr. Kontio, and Dr. Carsen).

## Abstract

Bilateral discoid medial menisci are exceptionally rare and of uncertain pathoetiology. We report on a case in an active adolescent woman who presented with bilateral medial knee joint line pain. Clinical evaluation and MRI identified atypical bilateral discoid medial menisci bilaterally. The patient underwent bilateral meniscal saucerization, with no pain at 4 months postoperation, and returned to competitive sport.

Bilateral discoid medial menisci (BDMM) represent an exceptionally rare congenital pathology of the knee joint that has low reported incidence.^1,2,3,4,5^ A discoid meniscus represents a meniscus with an atypical saucer-like shape that is thicker, covering a larger surface area of the tibial plateau compared with an anatomically normal crescent-shaped meniscus.^6,7^ Discoid menisci are markedly more common on the lateral side of the knee, with the first documented discoid medial meniscus reported by Cave and Staples in 1941^8^ and the first BDMM occurrence documented in 1956 by Murdoch.^4^

Since their discovery, controversy over the etiology involved in unilateral and bilateral discoid meniscus patterns have created many theories of their origin.^5^ In 1948, Smillie et al, hypothesized that the discoid menisci shape is a normal developmental stage during embryological development and that persistence of this shape, owing to failure of absorption of the central meniscus during the fetal stage, leads to “congenital discoid meniscus.”^8,9,10,11,12^ However, Weiner and Rosenberg (1974) provided evidence that as early as the 10th week of embryonic development, the cartilage of the knee has a shape that closely resembles that of an adult.^13-15^ Weiner and Rosenberg attributed the changes in the discoid meniscus to congenital alterations in the formation of the tibial plateau that then may alter the meniscus structure.^9,10,14-16^ Recent authors have also described flattening of the femoral condyles in the coronal plane of the knees concomitant with discoid medial meniscus, uncertain if the association is one of cause or effect.^12^ Kaplan (1957) believed that discoid menisci were not present normally at birth but developed over time because of an abnormal mechanical motion from the absence of the posterior tibial attachment.^12,16^ It was felt that the menisco-femoral ligament of Humphrey has an important role in discoid formation because the meniscus becomes hypermobile when there is an irregular attachment of the posterior coronary ligament.^3,6,11^ However, it must be noted that Kaplan mainly based his theory from the literature on discoid lateral menisci and not discoid medial menisci.^8^ In 2014, Raheel reported a case of familial discoid meniscus in which three members of a family presented with tears of their discoid medial meniscus, which lead to the theory of a genetic association with the etiology.^12^ In 2009, a histomorphological study by Papadopoulos et al,^17^ demonstrated discontinuity and nonhomogeneity of the circumferential collagen network in the discoid meniscus when compared with normal meniscus. The researchers proposed that these findings indicate that a discoid medial meniscus represents a structural lesion rather than a morphological variant,^17^ although no further information in the literature has confirmed this finding as of yet. Many of these theories focus on the etiology of unilateral discoid formations, with none specifically commenting on ideas of how a BDMM patterns form.

The incidence of BDMM is very hard to determine because of its rarity. A retrospective study conducted by Dickason et al (1982), where the researchers obtained data on 14,731 menisci from the Registry of Knee Surgery, of which 8040 were medial menisci. An analysis of these medial menisci found 10 were discoid (0.12%), with 1 patient (0.012%) having a BDMM pattern.^1,3,6,8-10,12,16,18,19^ A meta-analysis conducted by Marchetti et al (2007) based on the collective surveys of 35,000 medial menisci patients, of which 32 discoid medial menisci (0.089%) were identified. Of these 32 discoid medial menisci, four knees from two patients were acknowledged as having BDMM for an incidence of 0.0056%.^14^ Most of the literature references these two studies for the estimated range of the anomaly being between 0.0056% to 0.012%. However, the true rate of discoid medial menisci and the bilateral variant is likely higher because of its asymptomatic occurrence in most cases.^9^

Demographics of the discoid menisci population are evolving and are still somewhat unclear. There is an increased documented occurrence in Asian populations of Japan, India, and Korea compared with Caucasians, with rates reportedly up to 30% to 50% more frequent.^7,9,14,20^ However, these values do not separate the discoid lateral meniscus from the discoid medial meniscus or the bilateral patterns that can occur. For discoid medial menisci, there would seem to be a predilection for male patients in the symptomatic population, with Liu et al (2016) reporting their cases in 85.7% men.^20^ Furthermore, Liu et al (2016) identified that most reported symptomatic patients were younger than the age of 40 years,^20^ with many of the case reports published in individuals younger than 18 years of age.

Most discoid menisci occur exclusively on the lateral side of the knee,^4^ with reported incidence ranging from 0.4% to 1.7%.^4,12,18-20^ Unilateral medial discoid meniscus occurrence is estimated to be 0.06% to 0.3%.^3,4,12,20^ In the United States, estimated rates of discoid lateral meniscus have a narrower range of occurrence at 3% to 5% of the population, most which are predicted to be clinically silent.^18,19^

Discoid medial menisci are generally asymptomatic with most (60%) of the symptomatic presentations being atraumatic and insidious in onset.^2^ This would support the contention that their increased thickness and their potentially weakened posterior capsular attachment predispose to an increased likelihood of meniscus tearing, leading to symptomatic presentation,^5,21^ especially in athletic individuals.^2,5,14^ Symptomatic discoid medial meniscus can be associated with medial knee joint line tenderness, pain, effusion, locking, and stiffness, which can be the manifestation of associated meniscal tears, meniscal instability, secondary to abnormal mechanics because of the added and abnormal tissue in the medial compartment of the knee.^3,5,6,8^ BDMM can be hard to diagnosis becuase one knee is often asymptomatic.^5,9,20^ In addition, several anomalies have been found to be associated with discoid medial menisci, and these include tibial plateau depression, hypoplasia of the anterior horn attachment, and documented widening of the medial joint space on plain radiographs^3,9,10,12,21-23^ (Figure 1).

**Figure 1:**
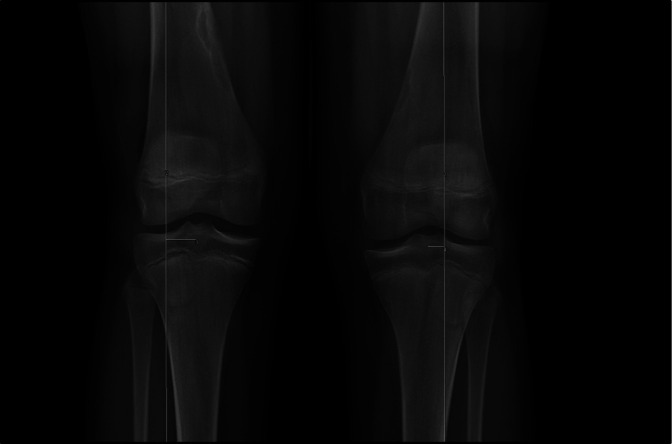
Anterior-posterior standing bilateral radiograph of the knees. Lateral mechanical axis deviation (MAD) and medial joint space narrowing. The patient was identified to have genu valgum bilaterally, with a MAD of 2.03 cm right and 1.03 cm left. Some relative widening of the medial joint space can also be appreciated.

This case report describes the diagnosis and treatment of symptomatic BDMM in a 14-year-old active girl who presented clinically with increasing bilateral knee tenderness and pain that began nine months earlier.

## Case

A 14-year-old female competitive hockey and softball player was referred for reports of progressive bilateral medial knee pain that developed atraumatically over 9 months. The patient had no issues weight-bearing but reported medial joint line pain bilaterally. Squatting, kneeling, running, twisting, and cutting movements aggravated her pain, obtaining relief on cessation of activities. The patient described a regular sensation of popping and locking within her right knee on a daily basis. The lateral menisci bilaterally were asymptomatic.

**Figure 2:**
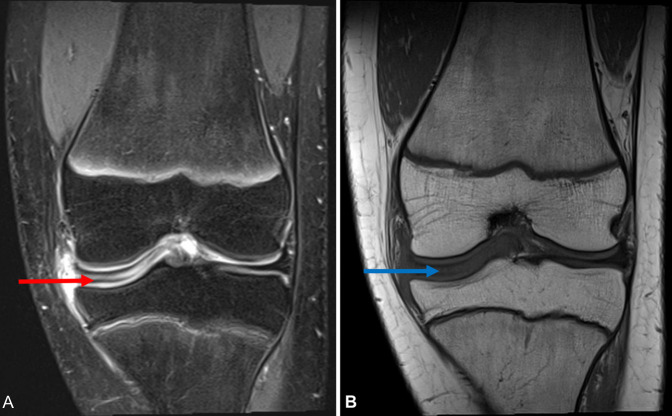
Coronal T2-weighted (**A**) and T1-weighted (**B**) MRI sections of patients left knee. Red arrow identifying the discoid medial meniscus of the T2-weighted image and blue arrow identifying discoid meniscus on T1-weighted image. A large horizontal tear extending from the posterior to the anterior meniscal horns with adjacent anterolateral parameniscal cysts was identified on this knee.

**Figure 3:**
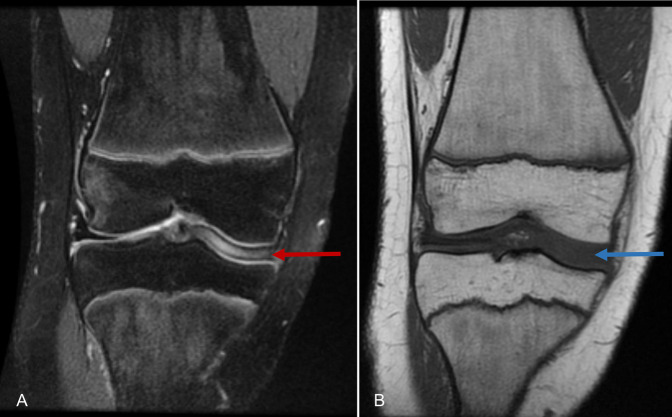
Coronal T2-weighted (**A**) and T1-weighted (**B**) MRI sections of the right knee. Red arrow indicating discoid medial meniscus on T2-weighted image, and blue arrow indicating discoid medial meniscus on T1-weighted image. Enlarged saucer-like appearance to the medial meniscus extending to the intercondylar notch was identified on the right knee in keeping with radio graphical diagnosis of discoid meniscus.

**Figure 4:**
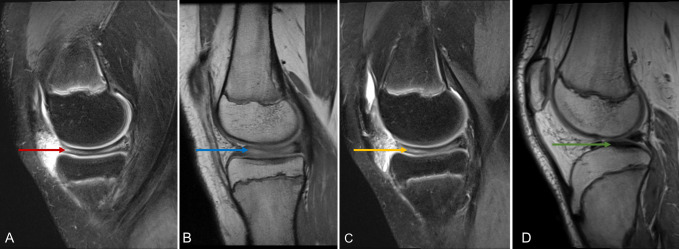
T2-weighted (**A** and **C**) and T1-weighted (**B** and **D**) sagittal MRI sections of the right (**A** and **B**) and left (**C** and **D**) knees. The red arrow identifies discoid medial meniscus on T2-weighted MRI section of right knee and blue arrow identifies the same discoid meniscus on T1-weighted section. The yellow arrow identifies discoid medial meniscus on T2-weighted section of left knee, with the green arrow marking the same meniscus of the left knee on a T1-weighted MRI section.

Physical examination revealed no knee joint effusion but displayed medial joint line tenderness bilaterally. The left knee showed slight reduced range of motion (10° to 120°) compared with the normal values in the right knee, with pain occurring at end range bilaterally. MRI of both knees demonstrated enlarged saucer-like appearance of the medial meniscus in the coronal plane, extending into the intercondylar notch, in keeping with a complete discoid meniscus (Figures 2–4).

**Figure 5:**
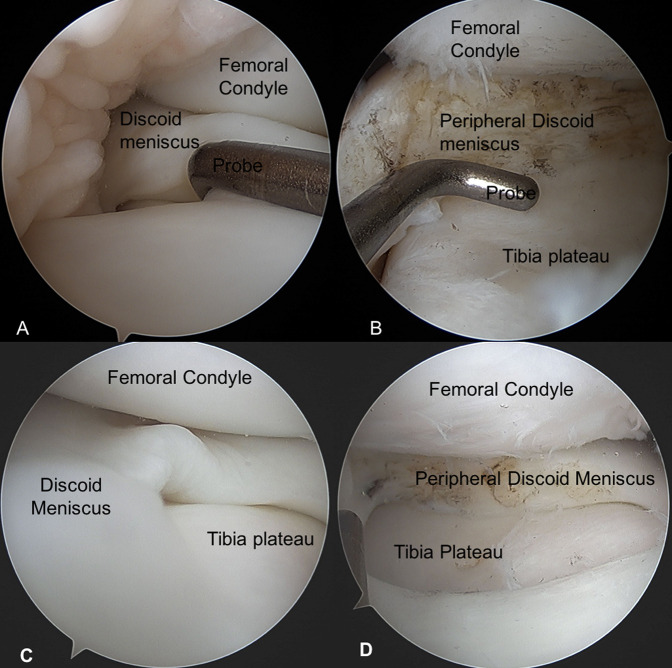
Intraoperative images of right knee and left knee. **A**, Arthroscopic image of discoid medial meniscus deformity with probe before surgical intervention of the right knee. **B**, Arthroscopic image postsurgical intervention of saucerization and caramelization of discoid meniscus, revealing the tibia plateau and remaining peripheral discoid meniscus of the right knee. **B** and **C** represents a meniscus more consistently with normal knee joint anatomy, although our patients postsaucerization. **C**, Arthroscopic image of in situ discoid meniscus of patient's left knee. **D**, Arthroscopic image of postsaucerization procedure of the left knee discoid meniscus. **B** and **C**, Postsaucerization and represent meniscus more consistent to normal knee joints. The meniscus in figure **B** is thicker than expected. The surrounding lateral compartment, notch, patellofemoral joint, and suprapatellar pouch were all normal bilaterally.

The patient underwent bilateral knee arthroscopy for complete discoid meniscus (Figure 5). Bilateral menisci were stable, but both were hypertrophic and complete discoid, the left knee's medial compartment being thinner and more mobile. Saucerization was performed of the central discoid element, essentially a partial meniscectomy centrally, until a stable remnant rim of meniscal tissue approximating a more normal meniscal width was reached.

## Discussion

Patel et al (1986), recommended conservative management of asymptomatic or minimally symptomatic patients and advocated for partial meniscectomy (ie, saucerization) of a symptomatic meniscus.^10,24^ Current preferred surgical management for symptomatic discoid meniscus is arthroscopic saucerization and peripheral suture repair for any associated meniscal tears.^3,4,6,19,25^

Saucerization reshapes the discoid meniscus with the removal of the central portion, creating a more normal crescent-shaped meniscus with an attempt at a more appropriate peripheral rim thickness^25-27^ (Figure 5). The saucerization procedure hypothetically restores the distribution of intra-articular force within the knee joint to that of an anatomically normal knee.^28^ Certain theories on the etiology of discoid meniscus speculate that symptoms develop from inappropriate mechanical stresses within the knee.^12,16^ Restoration of the knee joint to an anatomically normal structure, theoretically, should alleviate the patient's symptoms.^28^ Asymptomatic patients with confirmed discoid meniscus supports this theory because they indicate that the presences alone of the discoid meniscus does not cause symptoms, but mechanical stress put on the joint through usage develops the symptoms.^3,5,18,26,28,29^ Furthermore, the onset of cases with symptoms are documented earlier in younger athletic individuals than those leading sedentary lifestyles, indicating that the use of the joint may be part of the etiology.^5^

Saucerization alone does not correct for irregular attachments in the posterior coronary ligament or any associated anomalies, such as meniscus tears, that can present with discoid meniscus.^3,5,26,28,29^ However, long-term studies have identified statistically significant (*P* < 0.001) differences in the outcomes favoring saucerization over other treatment methods (ie, total meniscectomy) for discoid menisci.^28^ Identifying these patterns in studies supports the concept of meniscus preservation on the notion of redistribution of loading forces within the knee joint to relatively normal anatomic architecture can improve long-term outcomes for patients.^28,30^

Retrospective studies of this technique in the pediatric patient population demonstrate notable improvement in postoperative function and activity levels, with less degenerative changes over a 5-year period compared with total meniscectomy.^6,19,26,31^ Ahn et al (2015), analyzed the clinical outcome of discoid lateral menisci partial meniscectomy with or without suture repair to subtotal meniscectomy. Their findings revealed meniscus reshaping by partial meniscectomy was associated with decreased symptomatic degenerative changes compared with subtotal meniscectomy cohorts in long-term outcomes.^32^ Furthermore, Kim and Seo (2007) compared the 5-year radiological outcomes of partial meniscectomy to total meniscectomy for torn discoid lateral meniscus and found partial meniscectomies had less degenerative radiographic changes.^32^ No studies have been conducted of these surgical options with discoid medial meniscus because of the rarity of their occurrence.

Although partial meniscectomy, and repairs and stabilization where indicated, is the preferred management of discoid menisci, Ahn et al (2011) described somewhat unsatisfying results with the use of partial meniscectomy for treatment of lesions with horizontal cleavage or intrasubstance tear to the peripheral edge of the meniscus.^23,33,34^ They suggested that subtotal meniscectomy may be preferable in such cases.^33^ Furthermore, Wang^5^ (2018) reported retearing of the remaining discoid medial meniscus and secondary degenerative changes to the cartilage in the medial compartment of highly active young patients postpartial meniscectomy. Meniscal allograft transplantation for treating pain and improving knee function in these highly active patients with complicated follow-up postsaucerization has therefore been proposed.^5^ We feel a step-wise approach should be undertaken for each patient on an individual basis, with conservative management considered first and then progression to surgical intervention, with the continued aim of preservation of healthy meniscal tissue, wherever feasible.

## Conclusion

BDMM represent an extremely rare knee pathology with unclear pathoetiology and lon-term outcomes.^1,2^ Saucerization, and repair when necessary, represents the preferred surgical intervention for the treatment of symptomatic cases failing nonsurgical management. Consistently, favorable long-term outcomes, however, cannot be assured becuase some may present with future and ongoing injury and damage of joint structures. Future research on discoid medial menisci may be able to better clarify distinct and unique features, perhaps different from lateral discoid menisci, that will help guide optimum treatment.
